# Supplementary material for: Impact of the COVID-19 pandemic on emergency outpatient consultations and admissions of non-COVID-19 patients (ECCO)—A cross-sectional study
Source: PLoS One. 2022 Jun 10;17(6):e0269724. doi: 10.1371/journal.pone.0269724 (PMC9187104; doi:10.1371/journal.pone.0269724)
Supplement: S1 File — (PDF) [file pone.0269724.s001.pdf]

Questionnaire for general practitioners

1. Has there been a reduction/increase in your total workload as a general practitioner?
   If yes, by approximately what percentage?
   - Workload before lockdown = 100%
   - Load during lockdown: ___%
   - Load between 1st and 2nd wave: ___%
   - Load in 2nd wave: ___%

2. Please divide your workload before 1st lockdown and in 1st lockdown proportionally into the following areas: (in percentages, total should equal 100%).

- Consultation hours in the practice	before lockdown: ___%	in lockdown: ___%
- Home visits (excl. home care)	before lockdown: ___%	in lockdown: ___%
- Telephone consultations	before lockdown: ___%	in lockdown: ___%
- Organizational issues	before lockdown: ___%	in lockdown: ___%
- Home care	before lockdown: ___%	in lockdown: ___%
- Other...	before lockdown: ___%	in lockdown: ___%

3. Were you able to perform SARS-CoV-2 tests? Yes/No

4. In your opinion, did a shortage of care for chronically ill patients occur? Yes/No

5. Did patients present to physicians in poorer general health due to delays in visiting a physician?

6. Were there delays or cancellations of regular check-ups at the request of patients?

7. Were there any delays or cancellations of regular check-ups on your advice?

8. Were there patients who refused a recommended hospitalization due to the pandemic? Yes / No

9. If patients refused hospitalization, what were the reasons? (multiple answers possible)
   (Fear of infection, burden on health care system, risk situation, other?)

10. Did you tend to refrain from hospitalizations because of the pandemic? Yes / No

11. Did your patients experience an increase in mental health problems? Yes/No
    If yes, what mental health problems occurred? (Free text)

12. Were patient's advance directives created more frequently in the practice? Yes/No
    If yes, at the request of the patient or physician(s)?

Do you care for a nursing or retirement home(s)?   Yes/No

If yes, please also answer question 13 – 17 with reference to nursing home residents.

13. Were there any patients in the nursing/retirement home who declined recommended hospitalization due to the pandemic? Yes / No

14. If yes: Was this dependent on the underlying condition? (Cardiovascular, neoplasms, infectious, etc.)

15. What were the patients' reasons for refusal? (Fear of infection, burden on healthcare system, risk situation, other?)

16. Did you tend to refrain from hospitalizations due to the pandemic? Yes / No
    If yes: Was this dependent on the underlying condition? (Cardiovascular, neoplasms, infectious, etc.)

17. Were advanced directives created more frequently with patients in the nursing home? Yes/No
    If yes, at the request of the patients or as part of a concept of their institution?

In your opinion, are there any weaknesses in the pandemic strategy during the lockdown and suggestions for improvement for coming comparable situations? Are there any suggestions for improvement for the Cantonal Hospital Graubünden?

Questionnaire for nursing home administrators

1. How many patients do you care for in your institution?

2. How is medical care organized in your institution?
   - Home physician
   - former family physicians of the patients, who continue to care for them
   - mixed model

3. Were there patients who refused a recommended hospital admission due to the pandemic? Yes / No
   If yes: Was this dependent on the underlying condition? (cardiovascular, neoplasms, infectious).

4. Did you tend to refrain from consulting a physician for signs of illness due to the pandemic? Yes / No

5. If patients refused hospitalization, what were the reasons? (Multiple answers possible)
   (Fear of infection, burden on health care system, risk situation, other?)

6. Were advanced directives created with patients more frequently? Yes / No
   If yes, at the request of the patients or as part of a concept of their institution? (Free text)

7. Was the further procedure regarding hospitalization in case of a possible illness discussed with the patients more frequently? Yes / No
   If yes, at the request of the patients or within the framework of a concept of their institution? (Free text)

8. How many deaths occurred due to COVID-19 disease between the beginning of January 2020 and the end of January 2021 in your institution?

9. Has there been an increase in mental health problems due to the pandemic or contact restrictions? Yes/No
   If yes, what mental health problems occurred? (Free text)

10. Did your institution experience staff shortages due to quarantine/isolation, staff anxiety or burn-out?

Questionnaire for the internal medicine chief physicians of the referring hospitals

1. Did the 1st wave of the Corona pandemic result in a reduction or increase of admissions in your hospital? If reduction or increase, by approximately what percentage?

2. In the 2nd wave of the Corona pandemic, was there a reduction or increase in admissions in your hospital? If reduction or increase, by approximately how many percent?

3. In the 1st wave of the Corona pandemic, was there a reduction or increase in outpatient emergency medical consultations in your hospital?
   If reduction or increase, by approximately how many percent?

4. In the 2nd wave of the Corona pandemic, was there a reduction or increase in outpatient emergency medical consultations in your hospital?
   If reduction or increase, by approximately what percentage?

5. Were there any patients who were not referred to a center hospital because of the pandemic?
   1st wave: Yes / No
   2nd wave: Yes / No

6. If patients decided against referral: what were the reasons for refusal? (Fear of infection, burden on health care system, risk situation, other?)

7. Were advanced directives created more frequently with patients in your hospital? Yes / No
   If yes, at the request of the patients or as part of a concept of your hospital? (Free text)
