# Supplementary material for: Impact of the COVID-19 pandemic on emergency outpatient consultations and admissions of non-COVID-19 patients (ECCO)—A cross-sectional study
Source: PLoS One. 2022 Jun 10;17(6):e0269724. doi: 10.1371/journal.pone.0269724 (PMC9187104; doi:10.1371/journal.pone.0269724)
Supplement: S1 Table — BS1, BS2, BS3 denoted the basis splines, used in the non-linear model, depending on calendar week. Risk ratio (RR) 95% CI (confidence intervals) were reported for each predictor. (DOCX) [file pone.0269724.s003.docx]

**S1 Table.** Details of the statistical models (binomial negative) showed in Figures. BS1, BS2, BS3 are the basis splines used in the non-linear model depending on calendar week. Risk ratio (RR) 95 % CI (confidence intervals) were reported for each predictor.

|  | **All admissions** | | **Emergency** | | **Elective** | | **Emergency outpatient consultations** | |
| --- | --- | --- | --- | --- | --- | --- | --- | --- |
|  | **RR (95% CI)** | **p** | **RR (95% CI)** | **p** | **RR (95% CI)** | **p** | **RR (95% CI)** | **p** |
| **Calendar week** |  |  |  |  |  |  |  |  |
| BS1 (Week) | 0.41 (0.25, 0.68) | **<0.001** | 0.61 (0.37, 0.99) | **0.042** | 0.18 (0.07, 0.42) | **<0.001** | 0.50 (0.27, 0.90) | **0.020** |
| BS2 (Week) | 1.95 (1.40, 2.70) | **<0.001** | 1.58 (1.14, 2.18) | **0.005** | 2.42 (1.34, 4.35) | **0.003** | 1.37 (0.92, 2.05) | 0.123 |
| BS3 (Week) | 0.53 (0.40, 0.69) | **<0.001** | 0.67 (0.51, 0.88) | **0.003** | 0.33 (0.2, 0.55) | **<0.001** | 0.49 (0.35, 0.68) | **<0.001** |
| **Period (ref. Pandemic)** |  |  |  |  |  |  |  |  |
| Pre-pandemic | 0.95 (0.79, 1.16) | 0.628 | 0.94 (0.77, 1.13) | 0.491 | 0.92 (0.67, 1.25) | 0.576 | 1.02 (0.77, 1.36) | 0.867 |
| **Interaction Week:Period** |  |  |  |  |  |  |  |  |
| BS1(Week):pre-pandemic | 1.57 (0.88, 2.81) | 0.109 | 1.24 (0.70, 2.18) | 0.444 | 3.03 (1.15, 8.03) | **0.020** | 1.55 (0.67, 3.59) | 0.301 |
| BS2(Week): pre-pandemic | 0.59 (0.40, 0.85) | **0.005** | 0.65 (0.45, 0.94) | **0.021** | 0.54 (0.28, 1.05) | 0.069 | 0.84 (0.48, 1.47) | 0.546 |
| BS3(Week): pre-pandemic | 1.41 (1.02, 1.93) | 0.028 | 1.21 (0.89, 1.65) | 0.216 | 2.03 (1.15, 3.57) | **0.012** | 1.38 (0.87, 2.19) | 0.160 |
| **Goodness of fit** |  | **p** |  | **p** |  | **p** |  | **p** |
| Hosmer and Lemeshow test |  | 1.000 |  | 1.000 |  | 1.000 |  | 1.000 |

**Table S1**-continued

|  | **ICD Codes I** | | **ICD sub Codes I (I20-I24 vs I25)** | | **ICD Codes J** | | **ICD Codes C** | |
| --- | --- | --- | --- | --- | --- | --- | --- | --- |
|  | **RR (95% CI)** | **p** | **RR (95% CI)** | **p** | **RR (95% CI)** | **p** | **RR (95% CI)** | **p** |
| **Calendar week** |  |  |  |  |  |  |  |  |
| BS1 (Week) | 0.27 (0.15, 0.5) | **<0.001** | 0.23 (0.09, 0.58) | **0.002** | 0.39 (0.11, 1.37) | 0.121 | 0.36 (0.16, 0.81) | **0.013** |
| BS2 (Week) | 2.61 (1.74, 3.92) | **<0.001** | 2.58 (1.36, 4.93) | **0.004** | 0.25 (0.11, 0.58) | **0.001** | 2.75 (1.55, 4.91) | **0.001** |
| BS3 (Week) | 0.37 (0.26, 0.52) | **<0.001** | 0.37 (0.22, 0.63) | **<0.001** | 1.49 (0.78, 2.87) | 0.177 | 0.48 (0.29, 0.78) | **0.004** |
| **Period (ref. Pandemic)** |  |  |  |  |  |  |  |  |
| Pre-pandemic | 0.92 (0.74, 1.15) | 0.470 | 0.82 (0.59, 1.15) | 0.245 | 0.95 (0.59, 1.53) | 0.820 | 0.86 (0.64, 1.18) | 0.349 |
| **Interaction Week:Period** |  |  |  |  |  |  |  |  |
| BS1(Week):pre-pandemic | 2.6 (1.32, 5.11) | **0.005** | 4.12 (1.45, 11.68) | **0.007** | 1.41 (0.33, 5.92) | 0.626 | 2.2 (0.86, 5.57) | 0.099 |
| BS2(Week): pre-pandemic | 0.41 (0.26, 0.65) | **<0.001** | 0.31 (0.15, 0.63) | **0.001** | 0.88 (0.33, 2.31) | 0.783 | 0.42 (0.22, 0.8) | **0.009** |
| BS3(Week): pre-pandemic | 2.07 (1.41, 3.05) | **<0.001** | 2.39 (1.31, 4.38) | **0.005** | 0.46 (0.22, 0.98) | **0.027** | 1.86 (1.07, 3.25) | **0.029** |
| **I20- I24 (ref.) vs. I25** |  |  |  |  |  |  |  |  |
| ICD Code I 25 |  |  | 0.52 (0.47, 0.56) | **<0.001** |  |  |  |  |
| **Goodness of fit** |  | **p** |  | **p** |  | **p** |  | **p** |
| Hosmer and Lemeshow test |  | 1.000 |  | 1.000 |  | 1.000 |  | 1.000 |
