# Supplementary figures and images for: Impact of the COVID-19 pandemic on emergency outpatient consultations and admissions of non-COVID-19 patients (ECCO)—A cross-sectional study
Source: PLoS One. 2022 Jun 10;17(6):e0269724. doi: 10.1371/journal.pone.0269724 (PMC9187104; doi:10.1371/journal.pone.0269724)

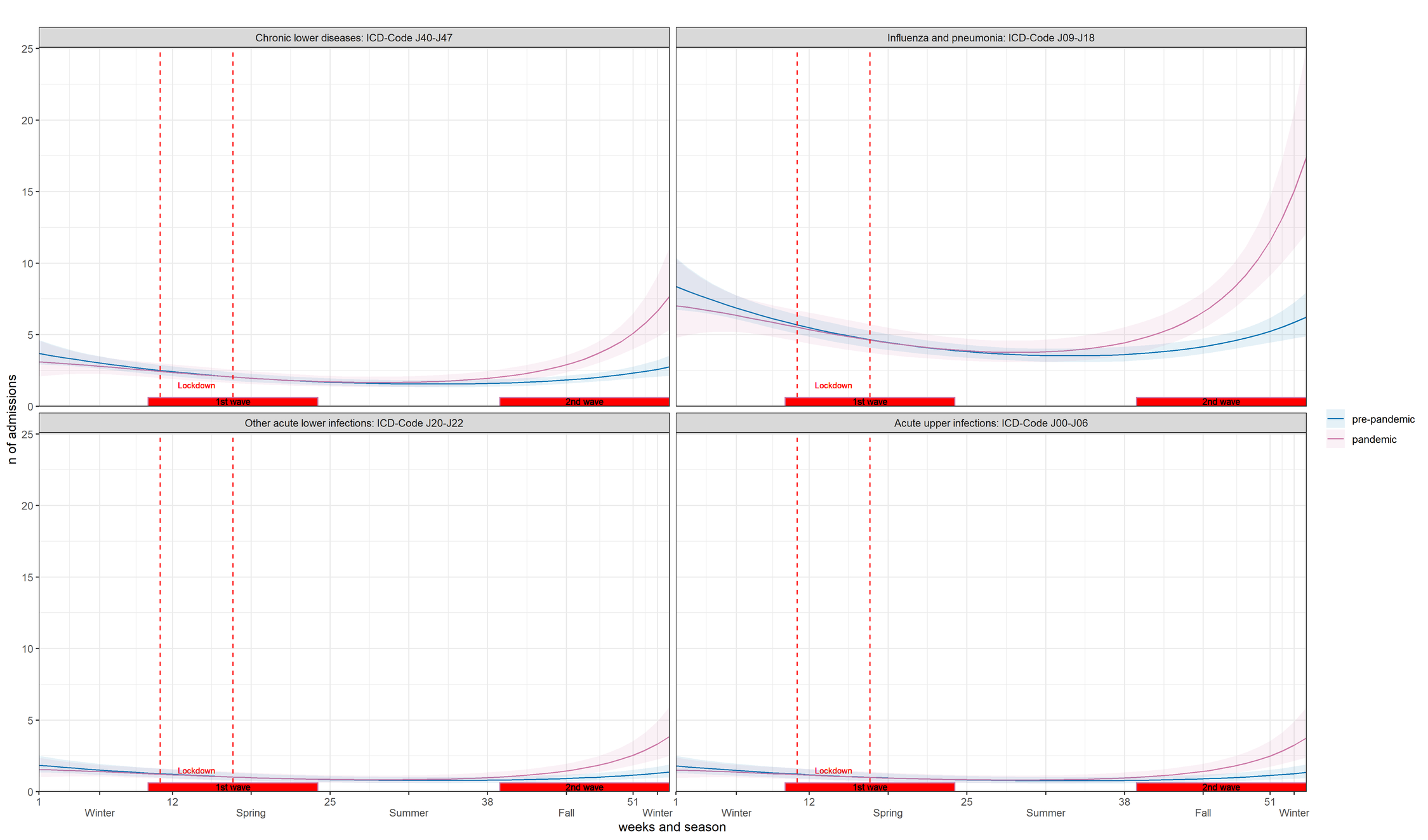

Supplement: S1 Fig — Negative binomial regression model of the number of admissions by International Classification of Diseases–10th (ICD) code. Subgroup analysis. J00-J06 (acute upper respiratory infections), J09-J18 (influenza and pneumonia), J20-J22 (other acute lower respiratory infections), J40-J47 (chronic lower respiratory diseases) 1st Wave: calendar weeks (10–24); 2nd Wave: calendar weeks (39–53). Lockdown: March 17—April 26. (TIF) [file pone.0269724.s004.tif]
